# Highly efficient biosynthesis of β-caryophyllene with a new sesquiterpene synthase from tobacco

**DOI:** 10.1186/s13068-022-02136-8

**Published:** 2022-04-25

**Authors:** Tao Cheng, Kai Zhang, Jing Guo, Qing Yang, Yiting Li, Mo Xian, Rubing Zhang

**Affiliations:** 1 CAS Key Laboratory of Bio-Based Materials, Qingdao Institute of Bioenergy and Bioprocess Technology, Chinese Academy of Sciences, Qingdao, 266101 China; 2 Tobacco Research Institute, Chinese Academy of Agricultural Sciences, Qingdao, 266101 China; 3 University of Chinese Academy of Sciences, Beijing, 100049 China

**Keywords:** β-Caryophyllene, *Escherichia coli*, Mevalonate pathway, Fed-batch fermentation, In situ extraction

## Abstract

**Background:**

β-Caryophyllene, a kind of bicyclic sesquiterpene, is mainly used as a spice in the food and cosmetic industries. Furthermore, it also has significant value in the pharmaceutical industry and is now considered to be used as a new fuel. As a chemical energy heterotrophic microorganism, *Escherichia coli* can produce a large amount of acetyl-CoA through aerobic respiration, and acetyl-CoA is the common precursor substance in the biosynthesis of all terpenoids. Therefore, *E. coli* has the potential to be a cell factory to produce terpenoids.

**Results:**

A new gene of β-caryophyllene synthase (TPS7) was found by analyzing the genome of *Nicotiana tabacum* L. using bioinformatics methods. The gene was overexpressed in engineered *E. coli* with a heterogeneous mevalonate (MVA) pathway to build a recombinant strain CAR1. Subsequent cultivation experiments in shake flask of engineered strain CAR1 verified that 16.1 mg/L β-caryophyllene was detected from the fermentation broth in the shake flask after induction for 24 h with IPTG. The toxic by-product of farnesyl acetate was detected during the process, and CAR1 showed a heavily cellular accumulation of product. We constructed an engineered strain CAR2, in which the downstream genes of the MVA pathway were integrated into the *E. coli* chromosome, successfully increasing β-caryophyllene production to 100.3 mg/L. The highest production of β-caryophyllene during the fed-batch fermentation was 4319 mg/L. Then we employed in situ extraction fermentation to successfully increase the production of β-caryophyllene by 20% to 5142 mg/L.

**Conclusion:**

A new sesquiterpene synthase, TPS7, from tobacco was found to be able to produce β-caryophyllene with high efficiency. Based on this, an engineered *E. coli* was constructed to produce a much higher concentration of β-caryophyllene than the previous studies. During the fermentation process, we observed that β-caryophyllene tends to accumulate in intracellular space, which will eventually influence the activity of engineered *E. coli*. As a result, we solved this by metabolism regulation and in situ extractive fermentation.

**Supplementary Information:**

The online version contains supplementary material available at 10.1186/s13068-022-02136-8.

## Background

Terpenes, the largest class of secondary metabolites in nature [1, 2], are mainly found in plants [3]. Terpenes play a crucial role in plant’s stress resistance, communication, and growth regulation [4]. β-Caryophyllene is a bicyclic sesquiterpene that was first identified in 1834, and has three conformations: *α*-, *β*- and *γ*-. Among them, the content of β-caryophyllene is the greatest in nature [5], which is typically employed as a fragrance or precursor of other more expensive scents [6], as well as a kind of potential aircraft fuel [7].

Generally, like other sesquiterpenes, the production of β-caryophyllene mainly relies on chemical synthesis and extraction from plants. Despite the excellent quality and natural activity, the plant extraction method is costly due to the low content in the plant and the complex multi-step extraction procedure. While the low cost of raw materials is a benefit [8], the complexity of the process and pollution of the environment push people to use microbial synthesis rather than chemical synthesis [9]. Compared to traditional methods, microbial synthesis will be a better choice due to its environmental friendliness and sustainability [10].

**Fig. 1:**
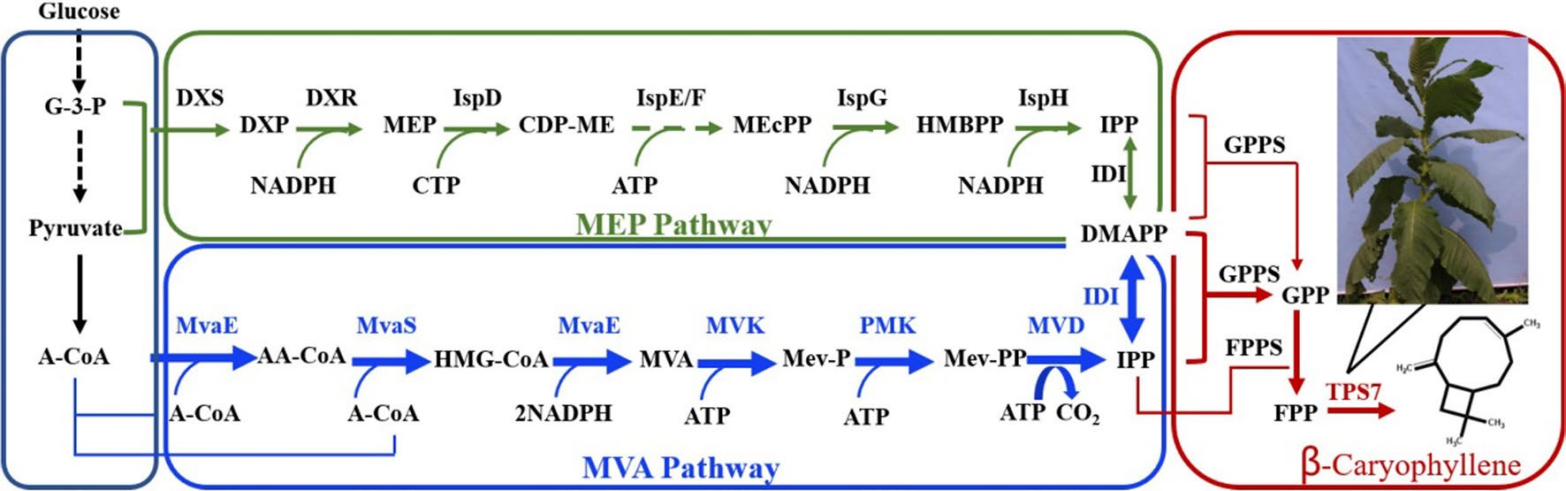
The heterologous MVA pathway introduced into *E. coli* to produce β-caryophyllene. Enzymes and some of the reaction intermediates necessary for the production of β-caryophyllene through the MVA pathway. Enzymes involved in the MVA and MEP pathway: *MvaE* acetyl-CoA acetyltransferase/HMG-CoA reductase, *MvaS* HMG-CoA synthase, *MK* mevalonate kinase, *PMK* phosphomevalonate kinase, *MVD* mevalonate pyrophosphate decarboxylase, *IDI* *IPP* isomerase, *DXS* 1-deoxy-D-xylulose-5-phosphate synthase, *DXR* 1-deoxy-D-xylulose 5-phosphate reductoisomerase, *IspD* 4-pyrophosphocytidyl-2-C-methyl-D-erythritol synthase, *IspE* 4-pyrophosphocytidyl-2-C-methylerythritol kinase, *IspF* 2-C-methyl-D-erythritol 2,4-cyclopyrophosphate synthase, *IspG* 4-hydroxy-3-methylbut-2-enyl pyrophosphate synthase, *IspH* 1-hydroxy-2-methyl-butenyl 4-pyrophosphate reductase, *GPPS* geranylgeranyl pyrophosphate synthase, *FPPS* farnesyl pyrophosphate synthase. Intermediates involved in the MVA and MEP pathway: *G-3-P* glyceraldehyde 3-phosphate, *DXP* 1-deoxy-D-xylulose 5-phosphate, *MEP* 2C-methyl-D-erythritol 4-phosphate, *CDP-ME* 4-diphosphocytidyl-2C-methylD-erythritol, *MEcPP* 2C-methyl-D-erythritol 2,4-cyclodiphosphate, *HMBPP* 1-hydroxy-2-methyl-2-(E)-butenyl 4-diphosphate, *A-CoA* acetyl-CoA, *AA-CoA* acetoacetyl-CoA, *HMG-CoA* 3-hydroxy-3-methylglutaryl-CoA, *Mev-P* mevalonate 5-phosphate, *Mev-PP* mevalonate 5-diphosphate, *IPP* isopentenyl pyrophosphate, *DMAPP* dimethylallyl pyrophosphate, *GPP* geranyl diphosphate, *FPP* farnesyl pyrophosphate

The biosynthesis of all sesquiterpenes relies on the same precursor of farnesyl diphosphate (FPP), which can be produced through the mevalonate (MVA) pathway or the methylerythritol 4-phosphate (MEP) pathway [11, 12]. Although the MEP pathway has a higher theoretical yield, the exogenous MVA pathway in *E. coli* produces terpenes more efficiently [13, 14]. The MVA pathway generates isopentenyl pyrophosphates (IPP) and dimethylallyl pyrophosphate (DMAPP) from acetyl-CoA, both of which are the common substrates for terpene biosynthesis [11]. The geranyl diphosphate synthase catalyzes IPP and DMAPP to geranyl diphosphate (GPP), and then GPP is catalyzed to FPP by farnesyl diphosphate synthase [15]. The FPP can be directly converted to sesquiterpenes by sesquiterpene synthase (Fig. 1) [2, 15, 16].

Many plant sesquiterpene synthases have been successfully identified, characterized, and cloned to construct engineered microorganisms for producing some compounds, such as α-farnesene, longifolene, bisabolene, β-sesquiphellandrene, and viridiflorol [17–23]. As well, β-caryophyllene has been reported to be synthesized in different engineered microorganisms. Reinsvold et al. constructed an engineered cyanobacterium*, Synechocystis*, in which the β-caryophyllene synthase gene from *Artemisia annua* was inserted, and IPP and DMAPP were provided by native MEP pathway, resulting in an approximately 494 ng·L^−1^·week^−1^ of β-caryophyllene [22]. Yeast was also used to produce β-caryophyllene, in which the optimized engineered strain achieved a concentration of 104.7 mg/L in shake-flask culture [24]. Yang et al. introduced a β-caryophyllene synthase from three different species of *A. annua*, *Arabidopsis thaliana,* and *Zea perennis*, respectively, into the engineered *E. coli* containing the exogenous MVA pathway. The final engineered strain with β-caryophyllene synthase from *A. annua* achieved the highest production of 1.52 g/L in the fed-batch fermentation condition [25]. However, the concentration is not high enough to be put into industrial application. Thus, in order to enhance the production to make the bioprocess feasible for industrial production, it is necessary to find a synthase with higher efficiency and optimize the metabolic pathway of β-caryophyllene by synthetic biology. Many tobaccos are rich in terpenes which are the source of the aroma compositions. As one of the major aroma compositions, β-caryophyllene is often rich in some tobaccos [26, 27]. Thus, tobaccos may have the potential to contain a high-activity β-caryophyllene synthase.

In this study, a new β-caryophyllene synthase gene was found in *Nicotiana tabacum L*., which could synthesize β-caryophyllene with remarkable efficiency in engineered *E. coli* containing the exogenous MVA pathway. Subsequently, the synthesis pathway of β-caryophyllene was optimized to enhance the β-caryophyllene production and reduce the accumulation of by-products. Finally, in situ extraction fermentation was employed to alleviate the intracellular accumulation of β-caryophyllene, thus significantly improving the yield of β-caryophyllene.

## Results and discussion

### Analysis of gene tps7 from tobacco

**Fig. 2:**
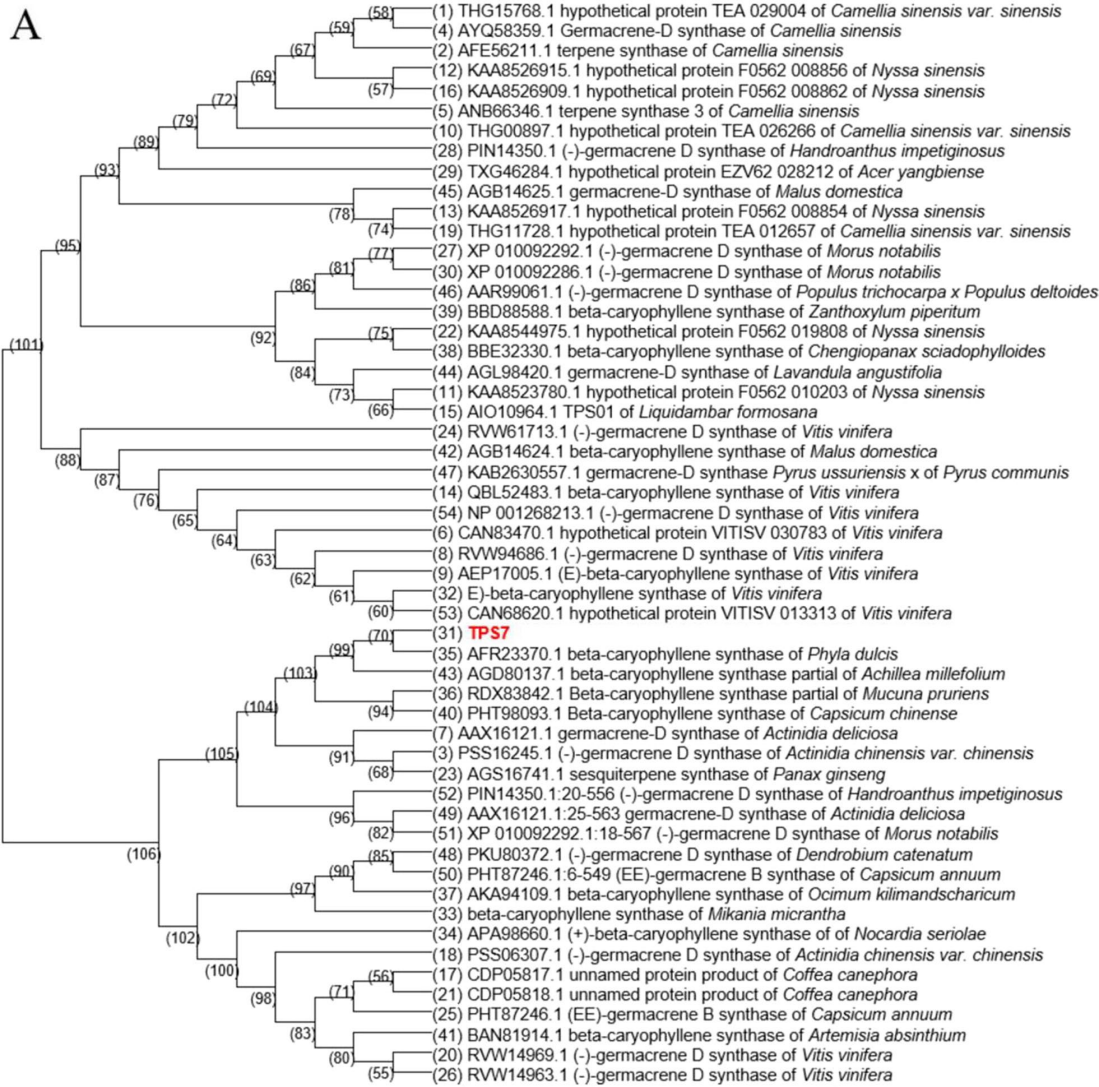

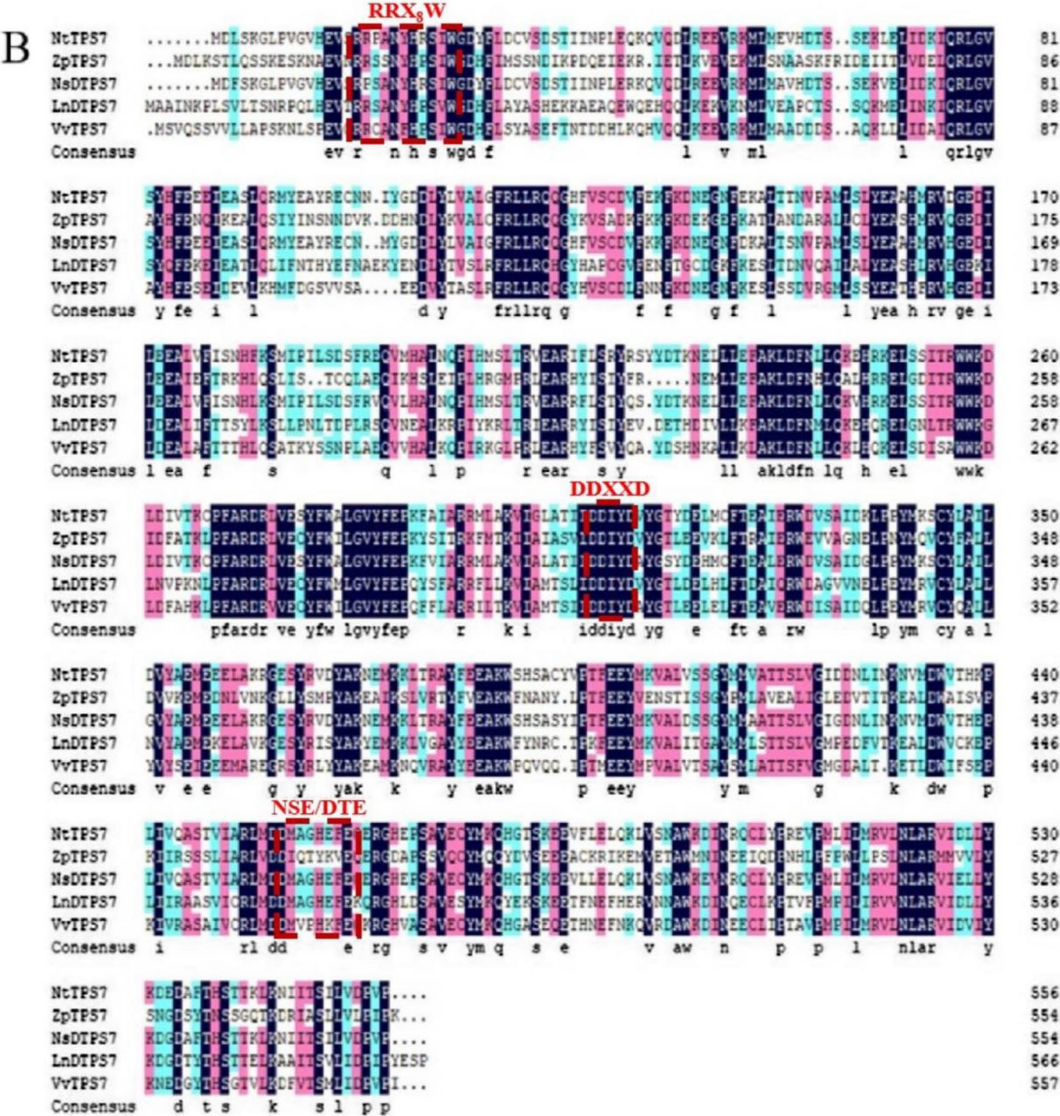
Bioinformatic analysis of sequence of protein and gene. **A** Evolutionary tree of *tps7* and genes of terpene synthase. **B** Sequence alignment of TPS7 and β-caryophyllene synthases from other species

**Fig. 3:**
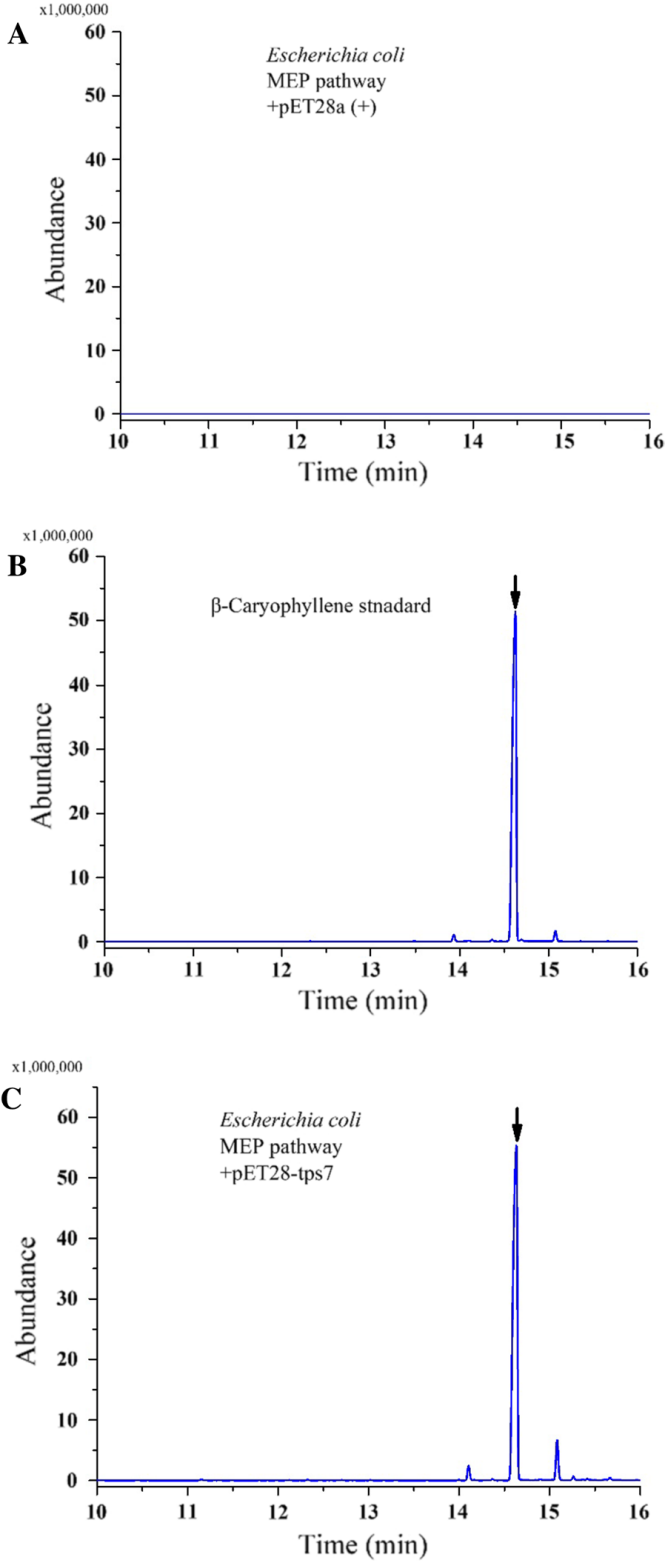

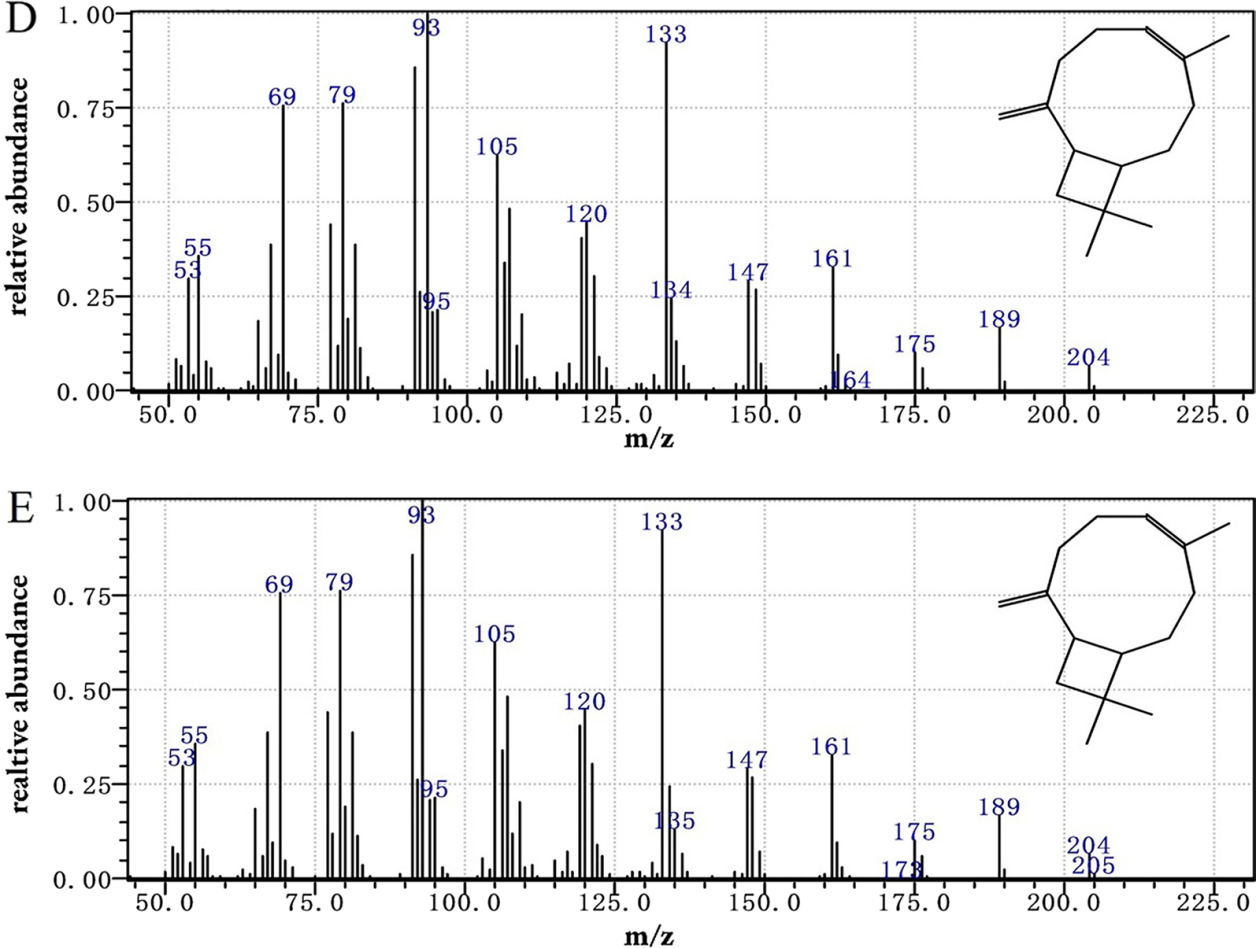
Analysis and identification of the product by GC–MS. **A** Total ion current chromatogram of the *E. coli* carrying pET-28a( +). **B** Total ion current chromatogram of the β-caryophyllene standard (the peak of β-caryophyllene was marked with an arrow corresponding to the retain time 14.6 min). **C** Total ion current chromatogram of the *E. coli* carrying pET-tsp7. **D** Mass spectra of the β-caryophyllene standard. **E** Mass spectra of the fermentation product

NtTPS7 was obtained by PCR amplification using *Nicotiana tabacum* L. cDNA as the template, according to the gene sequence from tobacco. The full-length 1671-bp gene sequence encoding 556 amino acids was obtained, and NCBI BLAST was used to align the gene and protein sequences. According to the results of NCBI BLAST, the amino acid sequence (Additional file 1: Table S1) of TPS7 from *Nicotiana tabacum L.* showed high similarity with the sequences of various sesquiterpene synthases. As shown in Figs. 2A and 3, the highest similarity was germacrene-D synthase, followed by β-caryophyllene synthase. The similarity of amino acid sequences was about 40%, and the similarity of synthases from various sources was significantly different. Furthermore, cluster analysis was performed on TPS7 and other sesquiterpene synthases with high sequence similarity from different sources to verify the evolutionary relationship. As shown in Fig. 1A, the evolutionary tree showed that TPS7 and β-caryophyllene synthase had a closer evolutionary relationship than other terpene synthases.

Similarly, the results of protein sequence alignment (Fig. 2B) showed that although the similarity between TPS7 and β-caryophyllene synthase was not the highest, there were many conserved sequences, especially in the area between C232 and G315. TPS7, like all other β-caryophyllene synthases, contains several highly conservative amino acid sequences, which are the terpene synthase active catalytic centers “DDXXD”, “RRX_8_W”, and “NST/DTE” [28, 29]. Therefore, we speculated that TPS7 might have the function of synthesizing β-caryophyllene.

### Expression and characterization of the TPS7 in E. coli

In general, terpene synthase from the plant was always poorly expressed in *E. coli* [30]*.* Therefore, to determine whether TPS7 can be normally expressed in *E. coli*, the gene was cloned into the prokaryotic expression vector pET-28a( +), resulting in the recombinant plasmid pET-tps7. The molecular weight of TPS7 is about 64 kDa. As shown in Additional file 1: Fig. S1, SDS-PAGE analysis showed that no substantial band matching the correct molecular weight was found in control *E. coli* carrying pET-28a( +). On the contrary, a specific band corresponding to the size of the target protein was visible in *E. coli* containing the plasmid pET-tps7, which showed that TPS7 without codon optimization could be successfully expressed in a soluble form in *E. coli.*

*E. coli* was able to maintain a small FPP pool, which was created via the native MEP pathway and served as a substrate for the catalysis of sesquiterpenoids synthesis [31]. To further characterize the biochemical functions of TPS7, the strain CAR0 (BL21/pET-tps7) and control *E. coli* carrying pET-28a( +) were cultured in shake flasks for accumulating target product. After incubation at 30 ℃ for 48 h, ethyl acetate was used to extract the culture broth. The upper organic phase was separated and used for gas chromatograph–mass spectrometry (GC–MS) analysis. The peak at 14.7 was confirmed to be β-caryophyllene by employing the relative retention time and mass spectra in comparison to an external standard. The peaks at 14.2 and 15.2 were identified as α-humulene and β-elemene by GC–MS, respectively, which were present in trace amounts. According to the standard curve, the β-caryophyllene titer reached 0.81 mg/L in a shake flask. This shows that the enzyme of TPS7 from tobacco is further identified as β-caryophyllene synthase.

To avoid confusion by isomerides of β-caryophyllene, we purified the crude product by TLC to get the pure product (Additional file 1: Fig. S2). Then the purified product was analyzed by NMR to affirm the actual structure. Comparing the analysis results of the purified product with the standard compound of β-caryophyllene (Additional file 1: Fig. S3), we found that the number and chemical shift of the two substances were absolutely the same so that the product could be identified as β-caryophyllene.

### Overexpression of exogenous MVA pathway in engineered E. coli to improved β-caryophyllene production

Although β-caryophyllene was produced utilizing the endogenous MEP pathway of *E. coli* and β-caryophyllene synthase, the native MEP pathway control mechanism restricts the supply of its precursor components IPP and DMAPP [32]. Recent investigations have demonstrated that the MVA pathway is more efficient for synthesizing IPP and DMAPP than the MEP pathway [14, 17]. As a result, a heterologous MVA pathway was introduced into *E. coli* to boost the production of the precursors IPP and DMAPP. In our previous work, the genes of the MVA pathway had been cloned into two plasmids, pACY-mvaE-mvaS which contains the genes of *mvaE*, *mvaS* from *Enterococcus faecalis*, and pYJM14 which contains the genes of *MVK*, *PMK*, *MVE*, and *IDI* from *Saccharomyces. cerevisiae* [33]*.* Subsequently, the *ispA* gene encoding GPP and FPP synthase and the *tps7* gene encoding β-caryophyllene synthase were cloned into the plasmid pACY-mvaE-mvaS, resulting in p-miT7. The strain CAR1 carrying the recombinant plasmids p-miT7 and pYJM14 was cultured in shake flasks to test its ability for β-caryophyllene production.

**Fig. 4:**
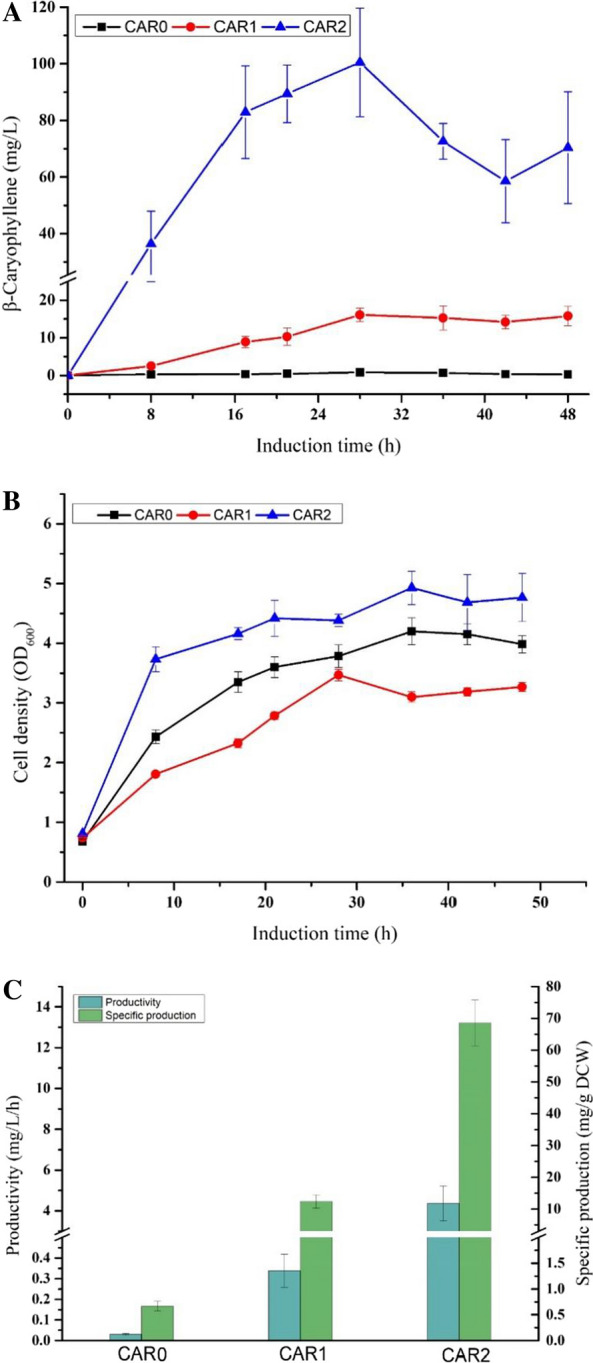
Comparison of the fermentation profiles between different strains in shake flask. **A** Time course profiles of β-caryophyllene accumulation; **B** time course profiles of Cell growth; **C** β-caryophyllene productivity and specific production of different engineered strains. The results represent the means ± S.D. of three independent experiments. Dry cell weight (DCW) was calculated according to the empirical formula: $\, 1{\text{ OD}}600 \, = \, 0.3 \, g{\text{ DCW}}/L$

The time-course profile of β-caryophyllene accumulation in the shake flask is shown in Fig. 4A. The β-caryophyllene titer of strain CAR1 with a heterogeneous MVA pathway reached 18 mg/L (15.4 mg/g DCW) after induction for 28 h, which was 22-fold higher than strain CAR0 with the native MEP pathway. However, as shown in Fig. 4B, the introduction of the MVA pathway negatively affected cell growth. The cell density of CAR1 overexpressing the MVA pathway was significantly lower than that of CAR0 with the origin MEP pathway during the cultivation process (Fig. 4B). We anticipated that the overexpression of the multi-gene pathway maybe resulted in the accumulation of intermediates, hence increasing the host’s metabolic burden. From detecting the fermentation broth of the shake flask, we found a series of by-products related to the biosynthetic process (Additional file 1: Fig. S4). Among these by-products, we identified one compound as farnesyl acetate, which is toxic to cells and inhibits DNA replication [34], synthesized from the substrate FPP. We speculated that the farnesyl acetate was produced due to the inherent low activity of β-caryophyllene synthase and the abundant availability of precursor FPP caused by MVA pathway overexpression. Nonetheless, these results also demonstrated that introducing the heterogeneous MVA pathway in *E. coli* could effectively increase the titer of β-caryophyllene.

### Optimization of β-caryophyllene biosynthetic pathways

Although introducing the heterogeneous MVA pathway in *E. coli* could improve β-caryophyllene production, the downstream operon in engineered strain CAR1 was initiated by a high copy number plasmid, resulting in a great amount of the intermediates, such as IPP, DMAP, and farnesyl acetate, which can be toxic to cells and lower the yield of the desired product. Compared to using free plasmids to produce desired products, chromosomal integrated metabolic pathways can improve the stability of foreign genes in cells, alleviate the metabolic burden on the host, and reduce the use of antibiotics in the production process [35]. In our prior work, a strain CM2 was created by integrating the *MVK*, *PMK*, *MVD*, and *IDI* genes into *E. coli* BL21(DE3) [36]. The plasmid of p-miT7 was transformed into strain CM2 to create a recombinant CAR2 for producing β-caryophyllene. The resulting strain was then tested for β-caryophyllene in shake flasks. Surprisingly, the shake-flask fermentation of CAR2 provided satisfactory results. Under the same cultivation conditions as CAR1, the β-caryophyllene titer of strain CAR2 was increased by sixfold in 24 h, from 16.1 to 100.3 mg/L, and the biomass of strain CAR2 improved by 1.5 times compared with strain CAR1 (Fig. 4A). The specific production and productivity of β-caryophyllene from CAR2 with the optimized MVA pathway reached 68.5 mg/g DCW and 4.3 mg/L/h, respectively, which was greater than that from CAR0 and CAR1 (Fig. 4C). The result was consistent with the previous studies that the integrated strain CM2 was beneficial for the production of terpenoids [36, 37]. Meanwhile, it was found that the concentration of farnesyl acetate was dramatically decreased (Additional file 1: Fig. S4). This result revealed that the appropriate down-regulation of the expression of the MVA pathway reduced the accumulation of intermediate metabolites for improving the yield of β-caryophyllene and the biomass, as well as alleviated the by-product accumulation.

### Intracellular or extracellular distribution of β-caryophyllene

**Fig. 5:**
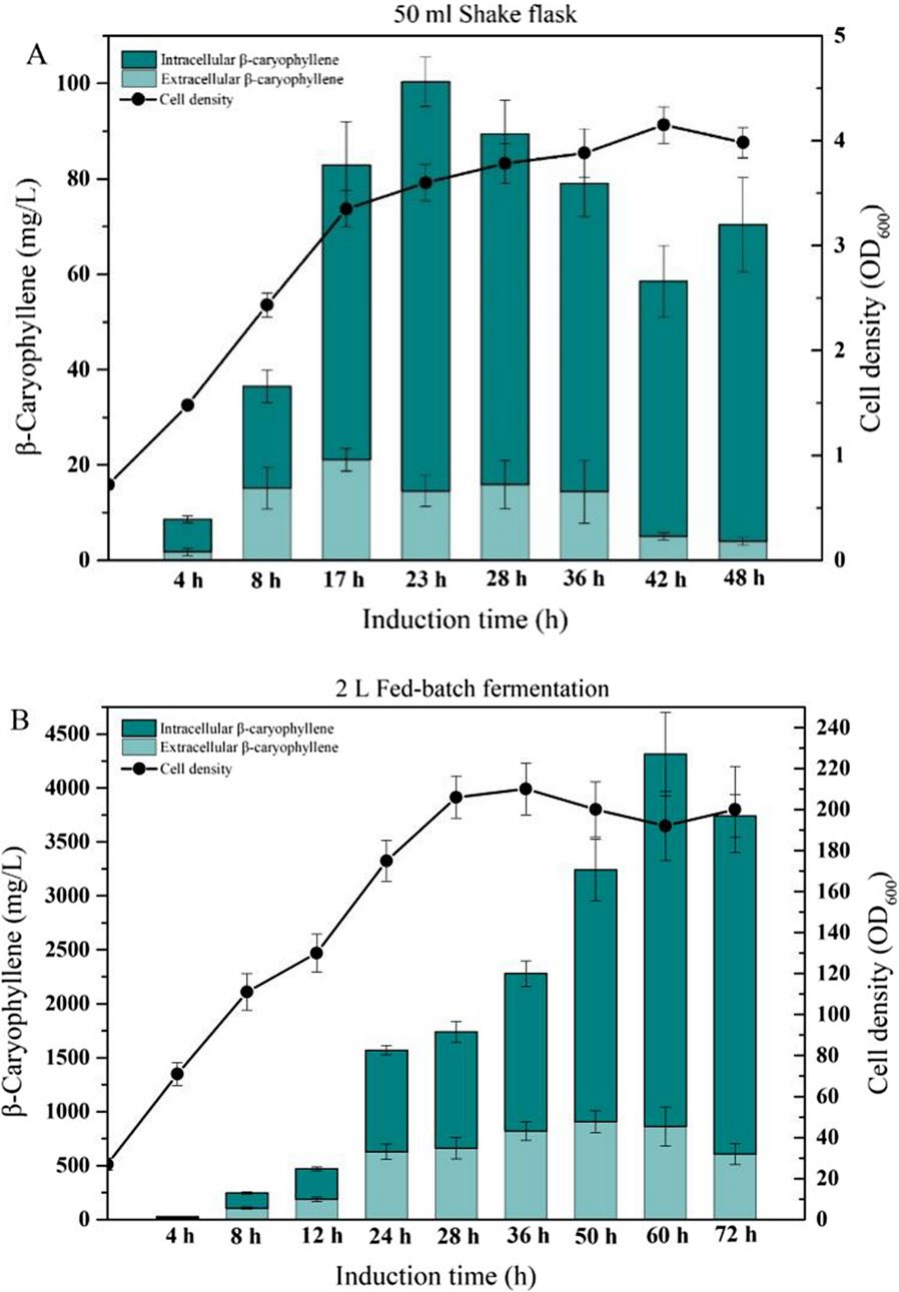
Time profiles of intracellular and extracellular distribution of β-caryophyllene in engineered strain CAR2. **A** β-caryophyllene titer in intra- or extracellular and cell density in shake flasks; **B** β-caryophyllene titer in intra- or extracellular and cell density in fed-batch fermentation

To investigate the distribution of β-caryophyllene in the shake flask, after 48 h of induction, the culture broth was centrifuged, and the cell and supernatant were extracted with ethyl acetate, respectively. For the first 23 h, 80 percent of β-caryophyllene was deposited in the intracellular space, and only 20% of the product was released into the culture broth, as shown in Fig. 5A. As the cultivation time goes by, the concentration of extracellular β-caryophyllene gradually decreases due to the volatility of β-caryophyllene. Only 5% of β-caryophyllene was detected in the extracellular after 42 h. Although most of the sesquiterpenoids are not suitable for intracellular accumulation due to their characteristics, such as cytotoxicity, evaporability, or secretion [38], our research showed that the β-caryophyllene primarily accumulated in the intracellular.

Subsequently, the culture volume was scaled up to 2 L in a 5-L bioreactor for fed-batch fermentation with the engineered *E. coli*. The cell growth rate in high-density cultivation was relatively stable and did not show significant fluctuation. Cell density grew stably during the fermentation process, which showed the engineered strain kept highly active in the synthesis process of β-caryophyllene. During the fermentation process of CAR2 (Fig. 5B), the production rate of β-caryophyllene was low in the first few hours and accelerated from about 4 h after induction. Then the product kept accumulating at high speed until the peak production, which appeared to be coupled with the cell growth and reached a highest concentration of 4319 mg/L with productivity of 67.5 mg/L/h in 60 h after induction. The maximum biomass reached an OD_600_ of 210 during the high cell density fermentation. Simultaneously, the intracellular or extracellular distribution of β-caryophyllene was determined. In contrast to shaking flasks (Fig. 5A), roughly 50% of the product is released into the extracellular medium during the early phases of fed-batch fermentation (4–24 h). After 24 h, it is consistently maintained at around 600 mg/L (Fig. 5B). However, with increasing cell density, the intracellular titer of β-caryophyllene gradually increased, resulting in a decrease in extracellular β-caryophyllene content to 16% and an increase in intracellular concentration to more than 80%. We anticipated that β-caryophyllene's solubility in water can readily exceed the threshold in an aqueous medium, resulting in most of the products in intracellular. Due to the excessive intracellular aggregation of β-caryophyllene, negative feedback regulation or changes in cell shape occur, preventing future product improvement.

**Fig. 6:**
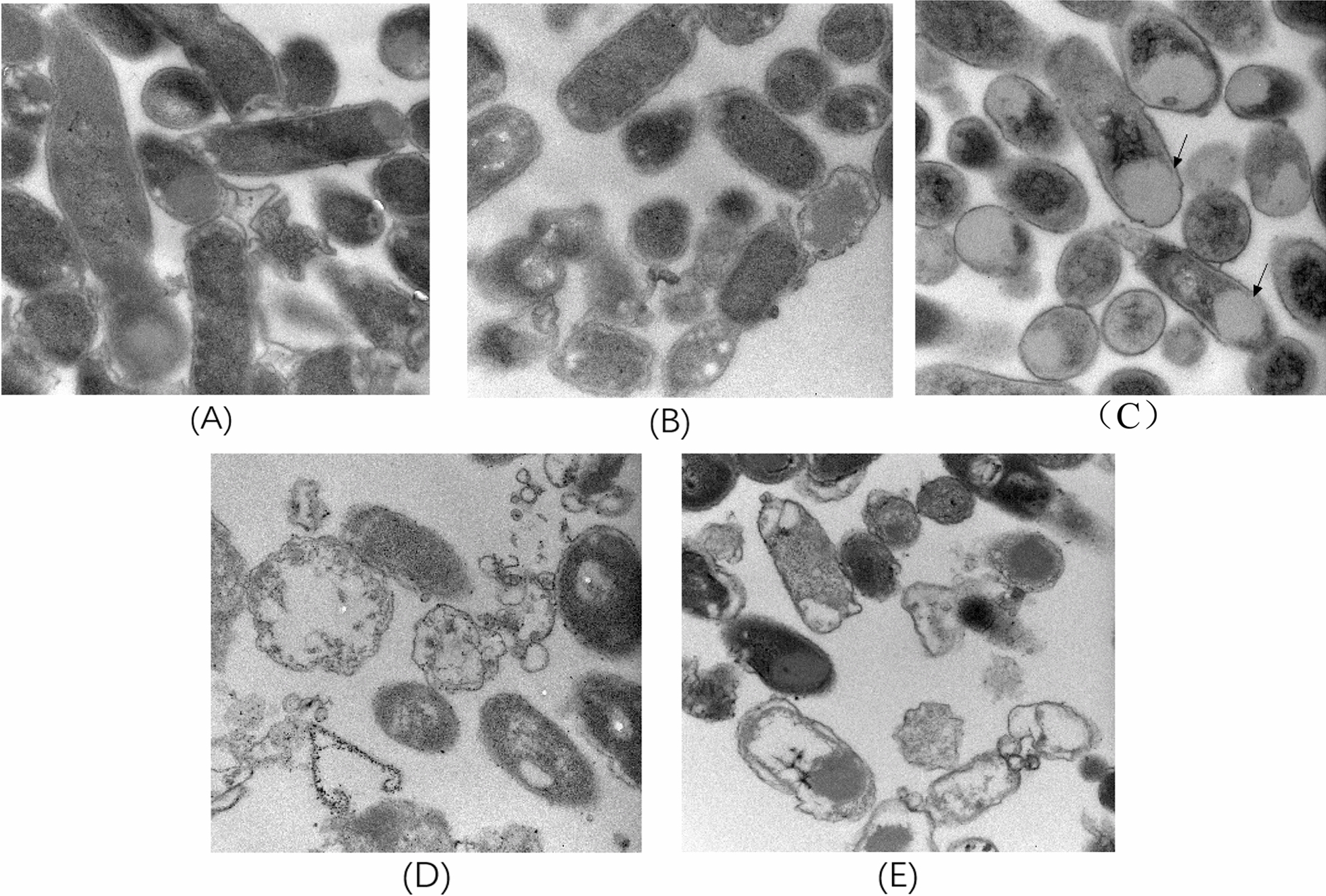
TEM observation of *E. coli* during fermentation process. **A** 0 h; **B** 12 h; **C** 24 h; **D** 36 h; **E** 48 h. Arrowpoint point to the droplet. Amplifications were × 40,000 for **A**, **B** and **C** and × 50,000 for **D** and **E**

To observe the changes in cell morphology with the accumulation of products in the intracellular, samples were collected and studied using transmission electron microscopy (TEM) at various points during the fermentation process (Fig. 6). Normal *E. coli* morphology was detected in the engineered strain CAR2 at 0 and 12 h with a normal rod shape, but a big white droplet has been observed in the cells after 24 h (Fig. 6C), which seems to cover about half of the cells, although the cell retains its native shape. However, some of the modified *E. coli* were almost filled with white droplets after 36 h, and the morphology became irregular or even ruptured to form cell fragments (Fig. 6D). This may be due to the excessive accumulation of β-caryophyllene in the intracellular, which causes damage to the cells, ultimately affecting the production of β-caryophyllene.

### In situ extraction fermentation

**Fig. 7:**
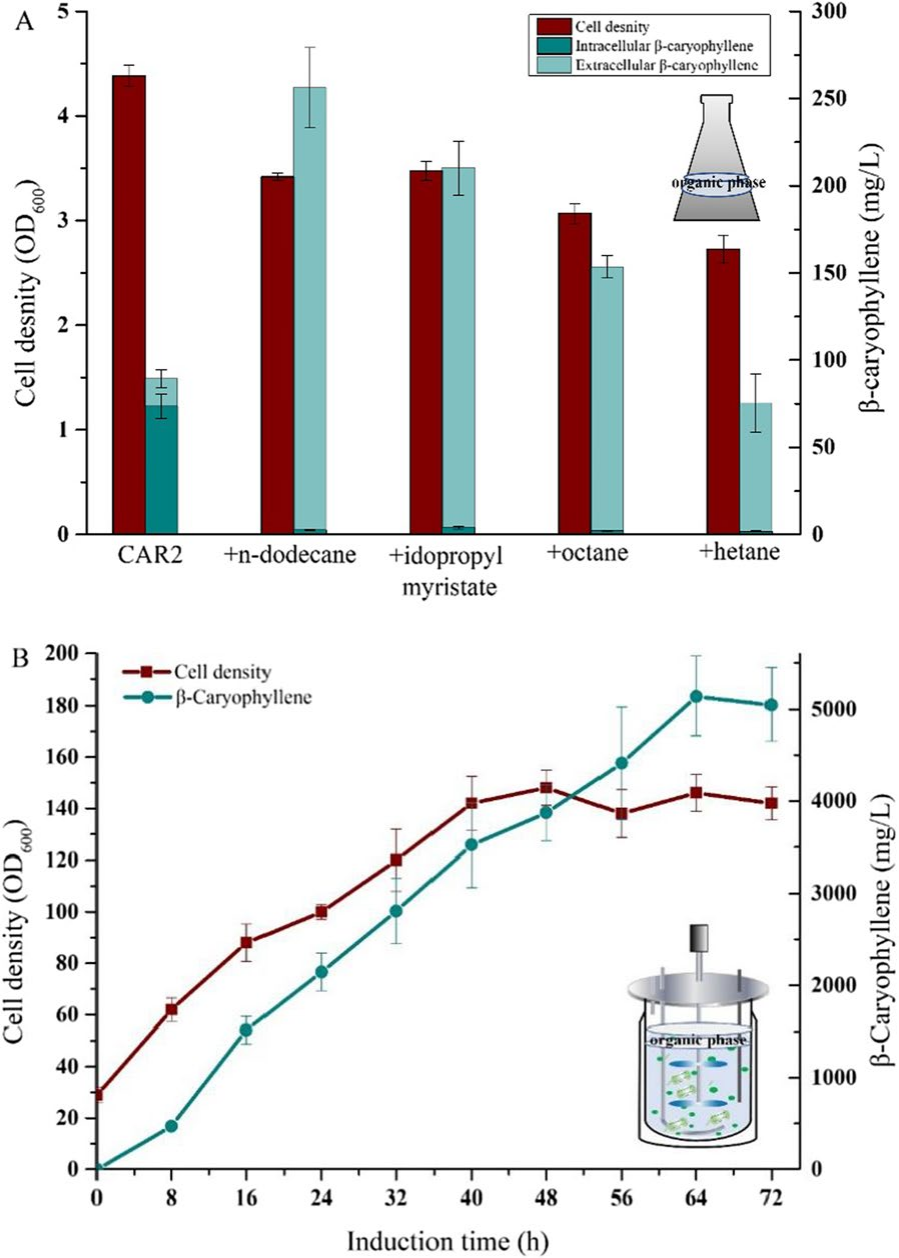
Effect of organic solvent on β-caryophyllene production. **A** Influence of different organic solvent in fermentation and Kinetics of cell growth and β-caryophyllene production during in situ fermentation of engineered strain CAR2 in shake flasks; **B** the time course of β-caryophyllene production and cell density during in situ fermentation

Most monoterpenoids or sesquiterpenoids are volatile and secreted outside the cells [39]. If the product is not excreted or only excreted in an insufficient quantity, then in situ extraction fermentation can be leveraged to boost or initiate product release due to cell permeabilization [40]. β-Caryophyllene has weak polarity and intense volatility, which may be responsible for product accumulating in the cells and escaping in a large amount. Therefore, the in situ extraction fermentation, in which the organic solvent was added into the fermentation broth for in situ extraction, was employed to extract products and preserve the product spillage. Based on this, the effects of organic solvent n-dodecane, octane, heptane, and isopropyl myristate on the production of β-caryophyllene and the cell growth of the engineered CAR2 were investigated. As shown in Fig. 7A, compared to the control group without an extraction agent, the β-caryophyllene titer of in situ extraction using n-dodecane, isopropyl myristate, octane, and heptane was 253, 206, 151, and 78 mg/L, representing 2.8-fold, 2.3-fold, 2.0-fold and 0.8-fold improvements, when compared with that achieved in no-solvent cultures (92 mg/L). The β-caryophyllene mainly accumulated in the extracellular (organic phase) and was barely detectable in the intracellular (Fig. 7A). Although the two-phase fermentation decreased the cell growth, the β-caryophyllene titer was significantly increased compared to the single-phase culture. It is verified that in situ extraction fermentation is useful for improving the yield of β-caryophyllene by relieving the end-product feedback repression. Due to the addition of n-dodecane having a significant effect on the improvements of β-caryophyllene titer and slight negative on the cell growth, we selected n-dodecane as the organic solvent in situ extractive fermentation.

In situ extraction was then used in the high-density fermentation of CAR2. The better-performed extraction agent, n-dodecane, was added into the fermenter with a ratio of 10% (v/v) at 2 h after induction. From the monitoring of the process (Fig. 7B), the addition of organic solvent increased the production of β-caryophyllene by 20%, effectively inhibiting the loss of β-caryophyllene. The highest β-caryophyllene titer reached 5142 mg/L with corresponding productivity of 80.3 mg/L/h in 64 h after induction, which was 2.7 times of the highest reported titer [25]. The maximum cell density reached 140 of OD_600_, a 30% decrease over the single-phase fermentation (210 of OD_600_). This result further showed that the addition of organic solvent adversely affected the growth of the strain [41].

In both shake-flask cultivation and fed-batch fermentation, in situ extraction fermentation enhanced the production of β-caryophyllene relative to single-phase fermentation. However, the β-caryophyllene titer just increased by 19% in fed-batch fermentation, which was significantly lower than the 2.5-fold increase rate in the shake-flask fermentation. It might be caused by the variation in cultivation conditions, such as in fed-batch fermentation, where the severe agitation and continuous aeration may be conceived to promote product secretion from intracellular to culture broth. Therefore, the percentage of β-caryophyllene in extracellular was maintained at approximately 20% during the fed-batch fermentation, whereas only 5% of β-caryophyllene was identified in the extracellular in shake flask (Fig. 5). The above results help to explain why adding organic solvent in the shake flask was more effective in enhancing the yield of β-caryophyllene than the fed-batch fermentation.

Correspondingly, the β-caryophyllene titer and cell density were just 1520 mg/L and 42 of OD_600_, respectively, in engineered *E. coli* under the fed-batch fermentation condition reported by Yang *et* al. [25]. Tracing back to the source, apart from the different sources of terpene synthase, this main reason is that the accumulation of toxic intermediates IPP and FPP leads to the imbalance of the MVA metabolic pathway, which was further confirmed by the comparison of cell density (OD_600_ of 210 Vs. OD_600_ of 40). To the best of our knowledge, this is the highest titer reported so far for β-caryophyllene in a microbial host [10, 22, 25]. Additionally, due to the addition of n-dodecane in the fermentation process, the β-caryophyllene accumulated nearly entirely in the organic phase, providing considerable convenience for the product separation and collection in the downstream processing. This finding indicated that the engineered strain had significant potential for industrial production of β-caryophyllene.

Despite the β-caryophyllene production in *E. coli* being hugely elevated by metabolic pathway optimization and in situ fed-batch fermentation, the conversion efficiency of glucose to β-caryophyllene (gram to gram) in the metabolically engineered strain is only 4.4% in the high cell density fermentation. In the future studies, several strategies will benefit the production and conversion efficiency of β-caryophyllene, such as: (1) at the level of enzyme engineering strategy, by altering critical amino acid residues involved in the catalytic function to enhance the activity of TPS7; (2) at the level of metabolic engineering strategy, by utilizing more potent promoters or raising the copy number to improve the expression level of TPS7 to limit the buildup of the intermediate metabolite FPP; (3) using a modular metabolic engineering strategy to separate the metabolic pathway into many modules, fine-tuning the expression level of inter and intra-modules to improve the metabolic flux [42]; (4) at the level of fermentation optimization strategy, the fermentation process can be monitored in real-time and online according to “Industry 4.0” [43] to collect and analyze significant amount of data from the metabolic process of the engineered strain, and take corrective actions to optimize the fermentation, resulting in further enhancing β-caryophyllene production.

## Conclusions

In this study, we found a novel β-caryophyllene synthase from tobacco with high activity of synthesis. Based on this, we constructed an engineered *E. coli* strain CAR2 that could produce β-caryophyllene with high efficiency. Through fed-batch fermentation and two-phase fermentation of engineered strain, the artificial synthetic pathway based on TPS7 can produce 5142 mg/L β-caryophyllene utilizing glucose as a carbon source, which is the highest production in the biosynthesis process by now. This finding indicated that the engineered strain had significant potential for industrial production of β-caryophyllene.

## Materials and methods

### Gene analysis

By genome sequence analysis of *Nicotiana tabacum L.*, several genes that may be related to terpene synthesis were found. These genes were analyzed by NCBI BLAST, and among these, a gene named *tps7* similarity with sequences of β-caryophyllene synthases was selected. From BLAST, genes that had similarities with *tps7* and had the function of terpene synthesis were all selected to draw an evolutionary tree with *tps7* by MEGA6.

Protein sequences of germacrene-D synthases and β-caryophyllene synthases from different species (ZpTPS7, GenBank: BBD88588; NsDTPS7, GenBank: APA98660; VvTPS7, GenBank: AEP17005.1; LnTPS7, GenBank: XP_019181134.1) were selected and used DNAMAN to test the similarity between TPS7 and these synthases.

### Plasmid and strain construction

All restriction enzymes used in this study were purchased from Thermo Fisher Scientific (Shanghai, China). PCR amplification was implemented using PrimeSTAR Max DNA Polymerase (TaKaRa, Beijing, China). All strains and plasmids used in this study are shown in Table 1. Primers used in this study were synthesized by GeneWiz (Suzhou, China) (Additional file 1: Table S2).

**Table 1:** Strains and plasmids used in the study

Name	Relevant genotype/property	Source
Strains		
*E. coli* BL21(DE3)	*F*^−^ *ompT hsdS*(*r*_*B*_^*−*^*m*_*B*_^*−*^) *gal dcm* (*DE3*)	TransGen Biotec
*E. coli* Trans5α	*F*^*−*^*φ80d lac ZΔM15 Δ(lacZYA-argF) U169 end A1 recA1 hsdR17(r*_*k*_^*−*^*, m*_*k*_^+^*) supE44λ- thi-1 gyrA96 relA1 phoA*	TransGen Biotec
CM2	BL21(DE3)::Trc-Low	[36]
CAR0	BL21(DE3)/pET-tps7	This study
CAR1	BL21(DE3)/pYJM14, p-miT7	This study
CAR2	CM2/p-miT7	This study
plasmids		
pET-28a( +)	oripBR322 lacI^q^ T7p, Kan^r^	Novagen
pEASY-tps7	pEASY-Blunt derivative carrying gene *tps7*, T7 promoter, Amp^R^, Kan^R^	Laboratory preserved
pYJM14	pTrcHis2B derivative carrying genes *MVK, PMK, MVE, and IDI*, Trc promoter, Amp^R^	[33]
pACY-mvaE-mvaS	pACYCDuet-1 derivative carrying genes *mvaE* and *mvaS*, T7 promoter, Cm^R^	[33]
p-miT7	pACYCDuet-1 derivative carrying genes *mvaE*, *mvaS*, *ispA* and *tps7*, T7 promoter, Cm^R^	This study
p-iT7	pACYCDuet-1 derivative carrying genes *ispA* and *tps7*, T7 promoter, Cm^R^	This study
pET-tps7	pET-28a( +) derivative carrying gene *tps7*, T7 promoter, Kan^R^	This study

Total RNA was extracted using the Plant Total RNA Kit (Nobelab Biotechnology Co. LTD, Beijing, China). The mRNA was purified by oligo dT-cellulose affinity chromatography using the FastPure^®^ Cell/Tissue Total RNA Isolation Kit V2 (TransGen Biotech, Beijing). A cDNA library was constructed using the Transcript Green One-Step qRT-PCR Supermix (TransGen Biotech, Beijing). The *tps7* gene was amplified from cDNA by PCR with primers and cloned into the plasmid pEASY-Blunt, resulting in pEASY-tps7.

The *tps7* gene and plasmid were amplified from pEASY-tps7 and pET-28a( +), respectively, and the two fragments were ligated using the pEASY-Blunt Zero Cloning Kit (TransGen Biotech, Beijing), resulting in the recombinant plasmid pET-tps7. Amplify the gene *tps7* from the plasmid pEASY-tps7 with primer tps7-F2 and tps7-R2 by PCR. The *ispA* gene was amplified from *E. coli* BL21(DE3) using primer ispA-F2 and ispA-R2. Gene segments of *tps7* and *ispA* were put into plasmid pACYCDuet-1, cut by the restriction endonuclease *Nde* I and *Xho* I, by homologous recombination, the recombinant plasmid was named p-iT7. Gene fragment mvaE-mvaS was amplified from vector pACY-mvaE-mvaS using primer MMG-F2 and MMG-R2, and put into plasmid p-iT7 cut by restriction enzyme *Nco* I and *Not* I to constitute plasmid p-miT7. Verified the recombinant plasmid with colony PCR using primers ispA-F2 and T7-ter and double digestion.

### Medium and culture conditions

Transform plasmid p-miT7 and pYJM14 into *E. coli* BL21(DE3) competent cell, pick out single colony and culture it with 5 mL LB medium (10 g/L tryptone, 5 g/L yeast extract, 10 g/L NaCl) with 50 μg/mL ampicillin and 34 μg/mL chloramphenicol overnight at 37 ℃, 200 rpm. Add 1 mL above culture into 50 mL M9 medium (15.3 g/L NaH_2_PO_4_·12H_2_O, 3 g/L KH_2_PO_4_, 1 g/L NH_4_Cl, 0.5 g/L NaCl, and adding 20 g/L glucose, 100 μL 1 M MgSO_4_ when using) with 50 μg/mL ampicillin and 34 μg/mL chloramphenicol, culture it at 37 ℃, 200 rpm, induce the fermentation broth with 0.3 mM isopropyl-β-D-thiogalactopyranoside (IPTG) when OD_600_ reaches 0.6, then keep on culturing for 24 h in 30 ℃.

### Fed-batch fermentation for β-caryophyllene biosynthesis

Fed-batch cultures for engineered *E. coli* were carried out in a 5 L glass fermenter (BIOSTAT B plus MO5L, Sartorius, Germany). The engineered *E. coli* was cultured for 8 h at 37 ℃ in 250-mL shake flask, filled with 100 mL LB medium as inoculum. The inoculum was added to 2 L fermentation medium (9.8 g/L K_2_HPO_4_·3H_2_O, 0.5 g/L yeast extract, 2 g/L MgSO_4_·7H_2_O, 2.1 g/L citric acid monohydrate and 0.3 g/L ferric ammonium citrate) supplemented with 20 g/L glucose, and 1 mL/L trace elements (3.7 g/L (NH_4_)_6_Mo_7_O_24_·4H_2_O, 2.9 g/L ZnSO_4_·7H_2_O, 24.7 g/L H_3_BO_3_, 2.5 g/L CuSO_4_·5H_2_O, 15.8 g/L MnCl_2_·4H_2_O). The temperature was kept at 37 ℃, and the 26% ammonia solution was added automatically to keep the pH at 7.0. The dissolved oxygen level was monitored and maintained at 30% saturation by adjusting the stirring speed. When the initial glucose was exhausted, keeping the stirring speed at 800 rpm, 50% glucose was added into the fermentation at a rate of 2–4.5 g/L/h to maintain the dissolved oxygen (DO) level at 40–50%. Once the cell density reached 20 of OD_600_, the fermentation system was then cooled to 30 ℃ and induced with 0.3 mM IPTG.

During in situ extraction fermentation, engineered *E. coli* was cultured under the same conditions, and organic solvent at a final concentration of 10% was added to the fermentation broth at 2 h after induction.

### Intracellular and extracellular distribution of β-caryophyllene

To investigate the intracellular and extracellular distribution of β-caryophyllene, the cell and supernatant were separated by centrifugation of 5 mL of fermentation broth, and the cellular precipitate was resuspended with 5 mL sterile water. An equal volume of ethyl acetate was added to the cell suspension and supernatant, respectively, and vortexed for 4 min to extract the product. Then, the mixture was centrifuged at 13,000 *g* for 2 min to obtain the organic phase with the desired product.

### TEM analysis of engineered E. coli

Solve 25.79 g Na_2_HPO_4_·12H_2_O and 4.37 g NaH_2_PO_4_·2H_2_O into 500 mL ddH_2_O as PB buffer, add 5 mL 50% glutaraldehyde into 50 mL PB buffer and fix the volume to 100 mL with ddH_2_O as 2.5% glutaraldehyde solution. The cultures were sampled at 0, 12, 24, 36 and 48 h, and the supernatant was discarded after centrifugation at 4000 rpm for 2 min. The cellular precipitate was resuspended and rinsed with 5 mL PB buffer, followed by fixation with 2.5% glutaraldehyde solution overnight at 4 ℃. The mixtures were centrifuged at 4000 rpm for 2 min, and the supernatant was removed. The obtained sample was rinsed twice with PB buffer and then analyzed with a TEM [44, 45].

### Purification and NMR analysis of β-caryophyllene

After fermentation, add 500 mL ethyl acetate into the broth, thoroughly mix it and centrifuge the mix at 10,000 rpm for 5 min, then collect all the organic phase and rotary evaporate the extract. Using TLC to purify the left liquid, load 5 mL raw product on the silica gel plate and develop with n-heptane. After the solvent reaching 1–2 cm away from the top of the gel plate, take out and dry the plate, then drop anisaldehyde at one side and heat at 200 ℃ for 5 min to determine the position of the product, collected the target band with the blade and crush it, solve the product with 10 mL ethyl acetate. Rotary evaporate the solution at 30 ℃ until there was no distillate and use a nitrogen blower to dry out the remaining liquid. Add 10 μL purified product into 500 μL chloroform-d, NMR analyzed the product.

^1^H-NMR and ^13^C-NMR spectra were recorded on Bruker NMR spectrometers (600 MHz). ^1^H-NMR chemical shifts (δH) and ^13^C-NMR chemical shifts (δC) are quoted in parts per million (ppm) downfield from trimethyl silane (TMS), and coupling constants (J) are quoted in Hertz (Hz).

### Analytical methods

Cell density was monitored by measuring the absorbance at 600 nm (OD_600_) with a spectrophotometer (Varian Cary 50 UV–Vis). During the fermentation processes, glucose concentration was determined using the YSI 2900 Biochemistry Analyzer.

For quantification and analysis of β-caryophyllene and by-products in the culture broth, the samples were added identical volume of ethyl acetate, vortexed briefly for 2 min, and centrifuged to separate the phases. Then the organic phase was filtered by a 0.22 μm filter membrane and analyzed by a GC–MS. The instrument was a Shimadzu GC-2010 Pro equipped with a triple–quadrupole mass spectrometer (TQ8050, Shimadzu, Kyoto, Japan). The GC equipped with an Rtx-5 ms capillary column (30 m × 0.25 mm × 0.25 μm, Restek, Bellefonte, PA, USA) was used to detect the product using an FID detector. The injection volume was 1 μL and the split ratio was 10:1 at 240 ℃. The N_2_ was used as the carrier gas with a linear velocity of 1 mL/min. The column temperature profile was 50 ℃ for 3 min, 10 ℃ /min increase to 290 ℃ and holding for 5 min. The injector and transfer line temperature were 240 and 250 ℃, respectively. The compound concentrations were calculated by interpolating with a standard curve prepared by standard β-caryophyllene. The mass spectrometer was operated in EI mode with complete scan analysis (m/z 50–230) [22, 25].

## Supplementary Information

**Additional file 1: Table S1.** Sequences of TPS7. **Table S2.** Primers used in this study. **Figure S1.** Protein expression analysis of TPS. **Figure S2.** GC analysis of purified product. **Figure S3.** NMR analysis of β-caryophyllene. **Figure S4.** GC analysis of farnesyl acetate.

## Data Availability

All data generated or analyzed during this study are included in this published article and its supplementary materials.

## Abbreviations

TPS7: β-Caryophyllene synthase
PTG: Isopropyl-β-d-thiogalactopyranoside
MVA: Mevalonate
MvaE: Acetyl-CoA acetyltransferase/HMG-CoA reductase
MvaS: HMG-CoA synthase
MK: Mevalonate kinase
PMK: Phosphomevalonate kinase
MVD: Mevalonate pyrophosphate decarboxylase
IDI: IPP isomerase
GPPS: Geranylgeranyl pyrophosphate synthase
FPPS: Farnesyl pyrophosphate synthase
MEP: 2C-Methyl-D-erythritol 4-phosphate
IPP: Isopentenyl pyrophosphate
DMAPP: Dimethylallyl pyrophosphate
FPP: Farnesyl pyrophosphate
DCW: Dry cell weight
GC: Gas chromatography
GC–MS: Gas chromatography mass spectrometry
OD_600_: Optical density at 600 nm
NMR: Nuclear magnetic resonance
TEM: Transmission electron microscopy
