# Supplementary material for: Highly efficient biosynthesis of β-caryophyllene with a new sesquiterpene synthase from tobacco
Source: Biotechnol Biofuels Bioprod. 2022 Apr 25;15:39. doi: 10.1186/s13068-022-02136-8 (PMC9040381; doi:10.1186/s13068-022-02136-8)
Supplement: Supplementary file 1 — Additional file 1: Table S1. Sequences of TPS7. Table S2. Primers used in this study. Figure S1. Protein expression analysis of TPS. Figure S2. GC analysis of purified product. Figure S3. NMR analysis of β-caryophyllene. Figure S4. GC analysis of farnesyl acetate. [file 13068_2022_2136_MOESM1_ESM.docx]

**Highly efficient biosynthesis of β-caryophyllene with a new** **sesquiterpene synthase from tobacco**

Tao Cheng^1†^, Kai Zhang^1†^, Jing Guo^1^, Qing Yang^2^, Yiting Li^2*^, Mo Xian^1,3^, Rubing Zhang^1,3^*

^†^These authors contributed equally to this work.

^1^ CAS Key Laboratory of Bio-Based Materials, Qingdao Institute of Bioenergy and Bioprocess Technology, Chinese Academy of Sciences, Qingdao 266101, China.

^2^ Tobacco Research Institute, Chinese Academy of Agricultural Sciences, Qingdao 266101, China.

^3^ University of Chinese Academy of Sciences, Beijing 100049, China.

* Correspondence:

Rubing Zhang

Mailing address: Qingdao Institute of Bioenergy and Bioprocess Technology, Chinese Academy of Sciences, Qingdao 266101, China

Email: zhangrb@qibebt.ac.cn

Yiting Li

Mailing address: Tobacco Research Institute, Chinese Academy of Agricultural Sciences, Qingdao 266101, China

E-mail: liyiting@caas.cn

## Table S1 Sequences of TPS7

| Name | Sequence |
| --- | --- |
| Amino acid sequence of TPS7 | MDLSKGLPVGVHEVSRRPANYHRSIWGDYFLDCVSDSTIINPLEQKQVQDLREEVRKMLMEVHDTSSEKLELIDKIQRLGVSYHFEEEIEASLQRMYEAYRECNNIYGDDLYLVALGFRLLRQQGHFVSCDVFEKFKDNEGNFEKALTTNVPAMLSLYEAAHMRVDGEDILEEALVFISNHFKSMIPILSDSFREQVMHALNQPIHMSLTRVEARIFLSRYRSYYDTKNELLLEFAKLDFNLLQKEHRKELSSITRWWKDLDIVTKCPFARDRLVESYFWALGVYFEPKFAIARRMLAKVIGLATIIDDIYDVYGTYDELMCFTEAIERWDVSAIDKLPPYMKSCYLAILDVYAEMEEELAKRGESYRVDYAKNEMKKLTRAYFEEAKWSHSACYVPTFEEYMKVALVSSGYMMVATTSLVGIDDNLINKNVMDWVTHKPLIVQASTVIARLMDDMAGHEFEQERGHEPSAVECYMKQHGTSKEEVFLELQKLVSNAWKDINRQCLYPREVPMLILMRVLNLARVIDLLYKDEDAFTHSTTKLKNIITSILVDPVP |
| Original gene sequence of *tps7* | ATGGATTTGAGCAAAGGCTTGCCGGTGGGAGTTCATGAAGTCTCTCGTCGCCCTGCAAATTATCATCGAAGCATTTGGGGAGACTATTTCCTTGATTGTGTTTCTGATTCCACGATTATTAATCCTCTGGAACAGAAACAAGTTCAAGACTTGAGAGAAGAAGTGAGGAAGATGTTAATGGAAGTCCATGACACGTCTTCAGAAAAGCTCGAGTTGATTGATAAAATCCAACGCTTAGGAGTATCATATCATTTTGAAGAGGAAATTGAGGCATCGCTACAAAGGATGTACGAAGCCTACCGTGAATGCAACAACATATATGGGGATGACCTTTATCTTGTTGCTCTTGGTTTTCGTTTACTAAGACAACAAGGCCATTTTGTATCTTGTGATGTGTTCGAAAAGTTCAAGGACAATGAAGGAAATTTTGAGAAGGCATTGACGACTAACGTGCCAGCAATGTTAAGTTTGTATGAAGCTGCACATATGAGAGTCGATGGAGAGGATATTCTCGAGGAAGCCTTAGTCTTCATATCAAATCATTTTAAATCAATGATTCCTATCTTGAGTGATTCCTTTAGGGAACAAGTAATGCATGCCCTAAATCAGCCAATCCATATGAGCTTAACAAGGGTAGAAGCAAGGATATTCCTATCTAGGTACCGAAGTTATTATGACACAAAGAATGAACTACTATTAGAATTTGCAAAGTTGGATTTCAACTTGTTGCAAAAGGAGCATAGGAAGGAGCTAAGTTCTATTACAAGGTGGTGGAAAGATTTGGACATTGTAACCAAGTGTCCTTTTGCACGAGATCGATTAGTAGAGAGCTATTTTTGGGCATTGGGAGTGTACTTTGAGCCAAAATTTGCTATTGCAAGAAGAATGCTCGCTAAAGTAATTGGCTTGGCTACCATTATCGACGACATCTACGATGTGTATGGAACTTACGATGAACTTATGTGTTTCACAGAGGCAATTGAGAGATGGGACGTTAGTGCCATTGATAAATTGCCGCCATACATGAAATCGTGTTATCTTGCCATTCTCGATGTTTATGCTGAAATGGAGGAGGAATTGGCCAAGAGAGGAGAATCTTATCGGGTTGACTACGCTAAAAATGAGATGAAAAAGTTGACTAGGGCATATTTTGAAGAGGCAAAGTGGTCTCATTCAGCTTGTTATGTCCCAACTTTTGAGGAGTACATGAAGGTTGCACTTGTTTCTAGTGGTTACATGATGGTTGCAACAACTTCTTTAGTTGGCATAGACGATAATTTGATAAACAAGAATGTCATGGATTGGGTTACACATAAACCTTTAATTGTTCAAGCTTCAACGGTAATTGCCAGACTGATGGATGACATGGCTGGACATGAATTTGAACAGGAAAGAGGACATGAACCTTCAGCAGTAGAGTGCTACATGAAGCAGCATGGAACATCAAAAGAAGAGGTGTTTTTGGAGCTTCAAAAATTAGTAAGTAATGCATGGAAAGATATAAACAGACAATGTTTGTATCCAAGAGAAGTACCAATGCTTATTCTCATGCGAGTTCTCAATCTTGCACGCGTGATAGATCTCCTTTACAAAGATGAAGATGCATTTACACATTCCACTACCAAGCTCAAGAACATCATTACTTCAATATTGGTTGATCCTGTTCCATAA |
| Optimized gene sequence of *tps7* | ATGGATCTGAGCAAAGGCCTGCCGGTGGGCGTGCATGAAGTGAGCCGCCGCCCGGCGAACTATCATCGCAGCATTTGGGGCGATTATTTTCTGGATTGCGTGAGCGATAGCACCATTATTAACCCGCTGGAACAGAAACAAGTGCAAGATCTGCGCGAAGAAGTGCGCAAAATGCTGATGGAAGTGCATGATACGAGCAGCGAAAAACTGGAACTGATTGATAAAATTCAGCGCCTGGGCGTGAGCTATCACTTCGAAGAAGAAATTGAAGCGAGCCTGCAGCGCATGTATGAAGCGTATCGCGAATGCAACAACATTTATGGCGATGATCTGTATCTGGTGGCGCTGGGCTTTCGCCTGCTGCGTCAGCAAGGCCATTTTGTGAGCTGCGATGTGTTTGAAAAATTTAAAGATAACGAAGGCAACTTTGAAAAAGCGCTGACCACCAACGTGCCGGCGATGCTGAGCCTGTATGAAGCGGCGCATATGCGCGTGGATGGCGAAGATATTCTGGAAGAAGCGCTGGTGTTTATTAGCAACCATTTTAAAAGCATGATTCCGATTCTGAGCGATAGCTTTCGCGAACAAGTGATGCATGCGCTGAATCAGCCGATTCATATGAGCCTGACCCGCGTGGAAGCGCGCATTTTTCTGAGCCGCTATCGCAGCTATTATGATACCAAAAACGAACTGCTGCTGGAATTTGCGAAACTGGATTTTAACCTGCTGCAGAAAGAACATCGCAAAGAACTGAGCAGCATTACCCGCTGGTGGAAAGATCTGGATATTGTGACCAAATGCCCGTTTGCGCGCGATCGCCTGGTGGAAAGCTATTTTTGGGCGCTGGGCGTGTATTTTGAACCGAAATTTGCGATTGCGCGCCGCATGCTGGCGAAAGTGATTGGCCTGGCGACCATTATTGATGATATTTATGATGTGTATGGCACCTATGATGAACTGATGTGCTTTACCGAAGCGATTGAACGCTGGGATGTGAGCGCGATTGATAAACTGCCGCCGTATATGAAAAGCTGCTATCTGGCGATTCTGGATGTGTATGCGGAAATGGAAGAAGAACTGGCGAAACGCGGCGAAAGCTATCGCGTGGATTATGCGAAAAACGAAATGAAAAAACTGACCCGCGCGTACTTTGAAGAAGCGAAATGGAGCCATAGCGCGTGCTATGTGCCGACCTTTGAAGAATATATGAAAGTGGCGCTGGTGAGCAGCGGCTATATGATGGTGGCGACCACGAGCCTGGTGGGCATTGATGATAACCTGATTAACAAAAACGTGATGGATTGGGTGACCCATAAACCGCTGATTGTGCAAGCGAGCACCGTGATTGCGCGCCTGATGGATGATATGGCGGGCCATGAATTTGAACAAGAACGCGGCCATGAACCGAGCGCGGTGGAATGCTATATGAAACAGCATGGCACGAGCAAAGAAGAAGTGTTTCTGGAACTGCAGAAACTGGTGAGCAACGCGTGGAAAGATATTAACCGTCAGTGCCTGTATCCGCGCGAAGTGCCGATGCTGATTCTGATGCGCGTGCTGAACCTGGCGCGCGTGATTGATCTGCTGTATAAAGATGAAGATGCGTTTACCCATAGCACCACCAAACTGAAAAACATTATTACGAGCATTCTGGTGGATCCGGTGCCGTAA |

## Table S2 Primers used in this study

| Primer | Sequence |
| --- | --- |
| mmG-F2 | 5’-TAATAAGGAGATATACCATGGCTAAAACAGTAGTTATTATTGATGCA  TTACG-3’ |
| mmG-R2 | 5’-CGACTTAAGCATTATGCGGCCGCTTAGTTTCGATAAGAGCGAA-3’ |
| ispA-F2 | 5’-TAAGAAGGAGATATACATATGGACTTTCCGCAGCAACTCGA-3’ |
| ispA-R2 | 5’-CCAGATTATTTATTACGCTGGATGATGTAGTCC-3’ |
| tps7-F2 | 5’-CAGCGTAATAAATAATCTGGTAAGGAGATATAATGGATTTGAGCAAA  GGCTTGC-3’ |
| tps7-R2 | 5’-GGTTTCTTTACCAGACTCGAGTTATGGAACAGGATCAACCAATATTG-3’ |
| 28a-F | 5’-GTTTAACTTTAAGAAGGAGATATACCATGGATTTGAGCAAAGGCTTGC  CG-3’ |
| 28a-R | 5’-ACGGAGCTCGAATTCGGATCCTTATGGAACAGGATCAACCAATATTG-3’ |
| T7-ter | 5’-GCTAGTTATTGCTCAGCGG-3’ |

## Figure S1 Protein expression analysis of TPS7

Recombine the fragment *tps7* amplified with primers 28a-F’ and 28a-R’ with plasmid pET-28a(+) which is cut by restriction enzyme *Nco* I *and Bam*H I, and transform the recombinant plasmid pET-*tps7* and empty plasmid pET-28a(+) into *E. coli* BL21(DE3) competent cell, then culture it with 50 mL LB culture medium with kanamycin in 30°C for 6h after induction. Collect the cell and disrupt the bacteria with high pressure, take 1 mL of the disrupted cell and add 1 mL 2 × loading buffer, heat in 100 °C for 5 min. Verified the sample by SDS-PAGE, using voltage 100 V in the whole course [1].

A new band present near 63 kD which conformed to the predicted molecular weight 64.5 kD indicated gene *tps7* could normally expressed in *E. coli*. Other bands were same as the blank group without gene *tps7*, which showed the expression of TPS7 did not influence the expression of other protein.


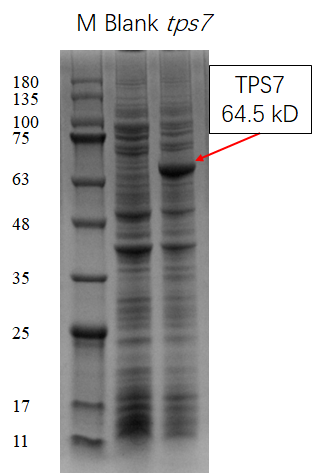


**Figure S1** Protein expression analysis of TPS7

## Figure S2 GC analysis of purified product

The crude product was separated into two bands on TLC, GC analysis showed one of the bands is the purified product which contained β-caryophyllene ratio of more than 95% [2, 3].


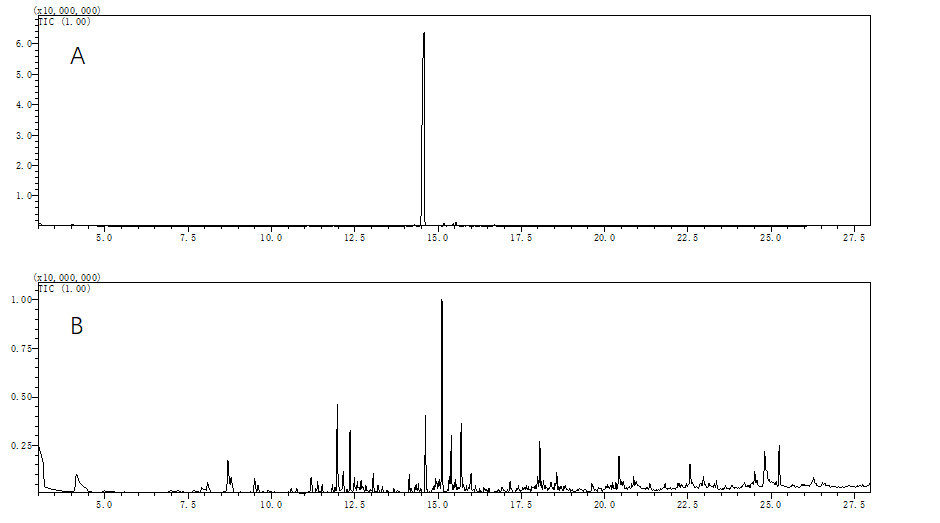


**Figure S2** GC analysis of different bands on TLC.

**A** The band of product; **B** The band of impurities.

## Figure S3 NMR analysis of β-caryophyllene

Add 10 μL purified product into 500 μL chloroform-d, NMR analyzed the product. ^1^H-NMR and ^13^C-NMR spectra were recorded on Bruker NMR spectrometers (600 MHz). ^1^H-NMR chemical shifts (δH) and ^13^C-NMR chemical shifts (δC) are quoted in parts per million (ppm) downfield from trimethyl silane (TMS), and coupling constants (J) are quoted in Hertz (Hz).


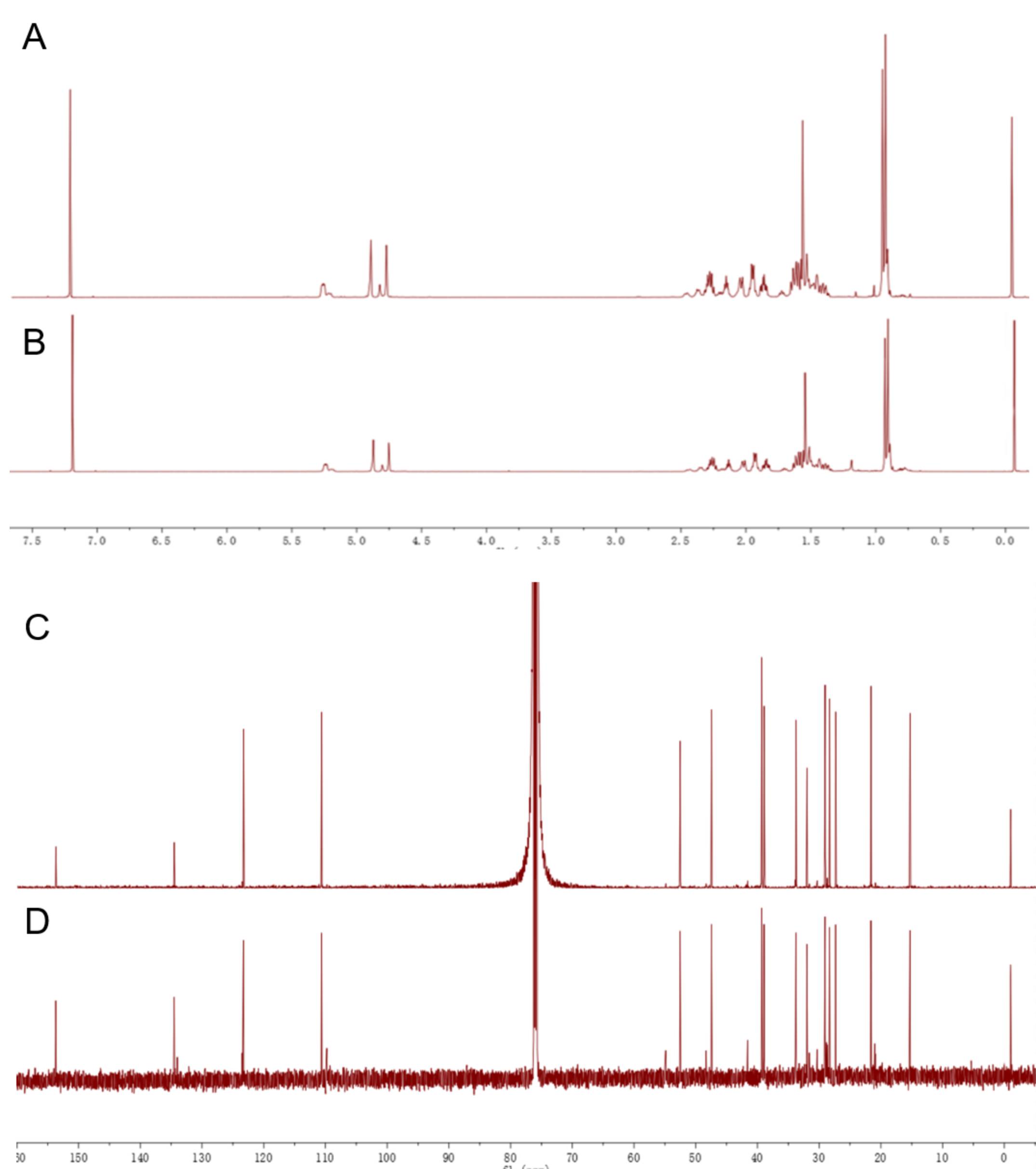


**Fig. S3** 600M NMR analysis of β-caryophyllene.

**A** ^1^H map of β-caryophyllene standard substance; **B** ^1^H map of purified product; **C** ^13^C map of β-caryophyllene standard substance; **D** ^13^C map of purified product

## Figure S4 GC analysis of by-product farnesyl acetate

The by-product was detected by GC, and its concentration in fermentation broth of different strain were compared through GC analysis software. The peak of by-product appeared at about 19.45 min.


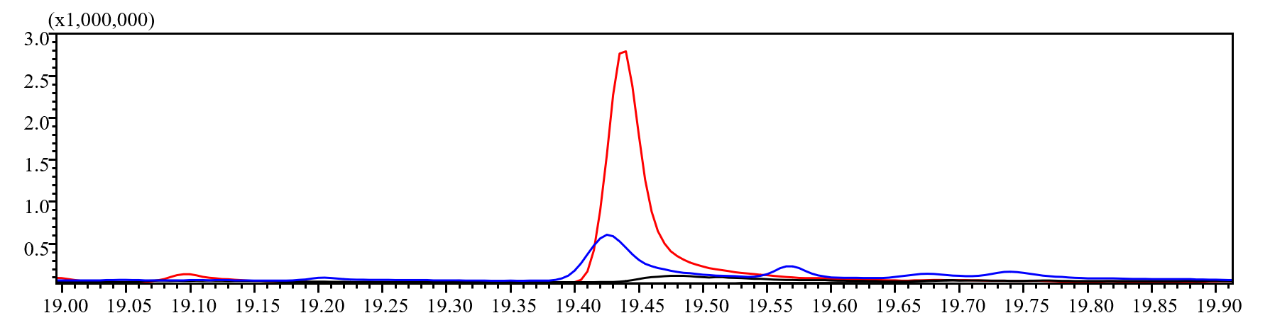


**Figure S4** GC analysis of farnesyl acetate.

**Black line**: strain CAR0; **Red line**: strain CAR1; **Blue line**: strain CAR2.

**Reference**

1. Muthusamy S, Vetukuri RR, Lundgren A, Ganji S, Zhu L, Brodelius PE, Kanagarajan S. Transient expression and purification of β-caryophyllene synthase in *Nicotiana benthamiana* to produce β-caryophyllene in vitro. Peerj. 2020;8:e8904.

2. Reinsvold RE, Jinkerson RE, Radakovits R, Posewitz MC, Basu C. The production of the sesquiterpene β-caryophyllene in a transgenic strain of the cyanobacterium *Synechocystis*. J Plant Physiol. 2011;168(8):848-52.

3. Yang J, Li Z, Guo L, Du J, Bae HJ. Biosynthesis of β-caryophyllene, a novel terpene-based high-density biofuel precursor, using engineered *Escherichia coli*. Renew Energ. 2016;99:216-23.
